# Towards high throughput GPCR crystallography: *In Meso* soaking of Adenosine A_2A_ Receptor crystals

**DOI:** 10.1038/s41598-017-18570-w

**Published:** 2018-01-08

**Authors:** Prakash Rucktooa, Robert K. Y. Cheng, Elena Segala, Tian Geng, James C. Errey, Giles A. Brown, Robert M. Cooke, Fiona H. Marshall, Andrew S. Doré

**Affiliations:** 1 Heptares Therapeutics Ltd, BioPark, Broadwater Road, Welwyn Garden City, Hertfordshire AL7 3AX UK; 2 Present Address: LeadXpro, Park InnovAARE, 5232 Villigen, Switzerland

## Abstract

Here we report an efficient method to generate multiple co-structures of the A_2A_ G protein-coupled receptor (GPCR) with small-molecules from a single preparation of a thermostabilised receptor crystallised in Lipidic Cubic Phase (LCP). Receptor crystallisation is achieved following purification using a low affinity “carrier” ligand (theophylline) and crystals are then soaked in solutions containing the desired (higher affinity) compounds. Complete datasets to high resolution can then be collected from single crystals and seven structures are reported here of which three are novel. The method significantly improves structural throughput for ligand screening using stabilised GPCRs, thereby actively driving Structure-Based Drug Discovery (SBDD).

## Introduction

Many of the world’s top selling drugs target G protein-coupled receptors (GPCRs)^1^ for indications including inflammaory, neurological, gastrointestinal, cardiovascular and respiratory diseases^2^. Structural data on this clinically relevant membrane protein superfamily has increased dramatically over the last decade, resulting from pioneering research from a number of groups^3,4^. High resolution crystal structures are now available for almost all major GPCR classes and are transformative from a pharmaceutical perspective, with several drug candidates generated by structure-based drug design (SBDD) techniques^5,6^. Nevertheless, GPCR crystallography throughput lags behind that of soluble targets (e.g. kinases)^7^, in part due to the inherent conformational flexibility and instability of GPCRs when removed from the native cell membrane environment. To overcome this, receptors have been thermostabilised by introducing a small number of targeted point mutations using the StaR^®^^8^, SABRE^9^ or CHESS^10^ technologies, or other mutagenesis approaches^11–13^. These mutations enhance apparent thermostability and stabilise receptors in a specific pre-defined conformation, and detergent-resistant form^14^. Such approaches were instrumental in solving structures of members of class B and C GPCRs^2,15,16^. Receptors stabilised using the StaR^®^ technology rely less on stability conferred by high affinity ligands to increase the chance of crystallogenesis. Co-crystal structures are thus obtainable even with low affinity compounds and fragments^17^ identified in early stages of discovery projects. This provides a unique opportunity to apply soaking techniques, successfully utilised for soluble targets (e.g. kinases), to GPCR crystals grown *in meso* by lipidic cubic phase crystallisation (LCP). The reliable production of multiple co-structures on a regular basis, in step with the medicinal chemistry cycle time, is fully enabling for SBDD.

Here, we report an *in meso* crystal soaking method developed to improve the crystallographic throughput for our work with the adenosine A_2A_ receptor (A_2A_R), including drug discovery activities. Previously, each ligand complex structure required a separate, bespoke A_2A_R-ligand protein preparation. Now a single protein preparation can yield high resolution structural data for A_2A_R in complex with up to a dozen different ligands. This also significantly minimises ligand amounts required to generate co-structures compared to using bespoke A_2A_R-ligand protein preparations.

**Figure 1:**
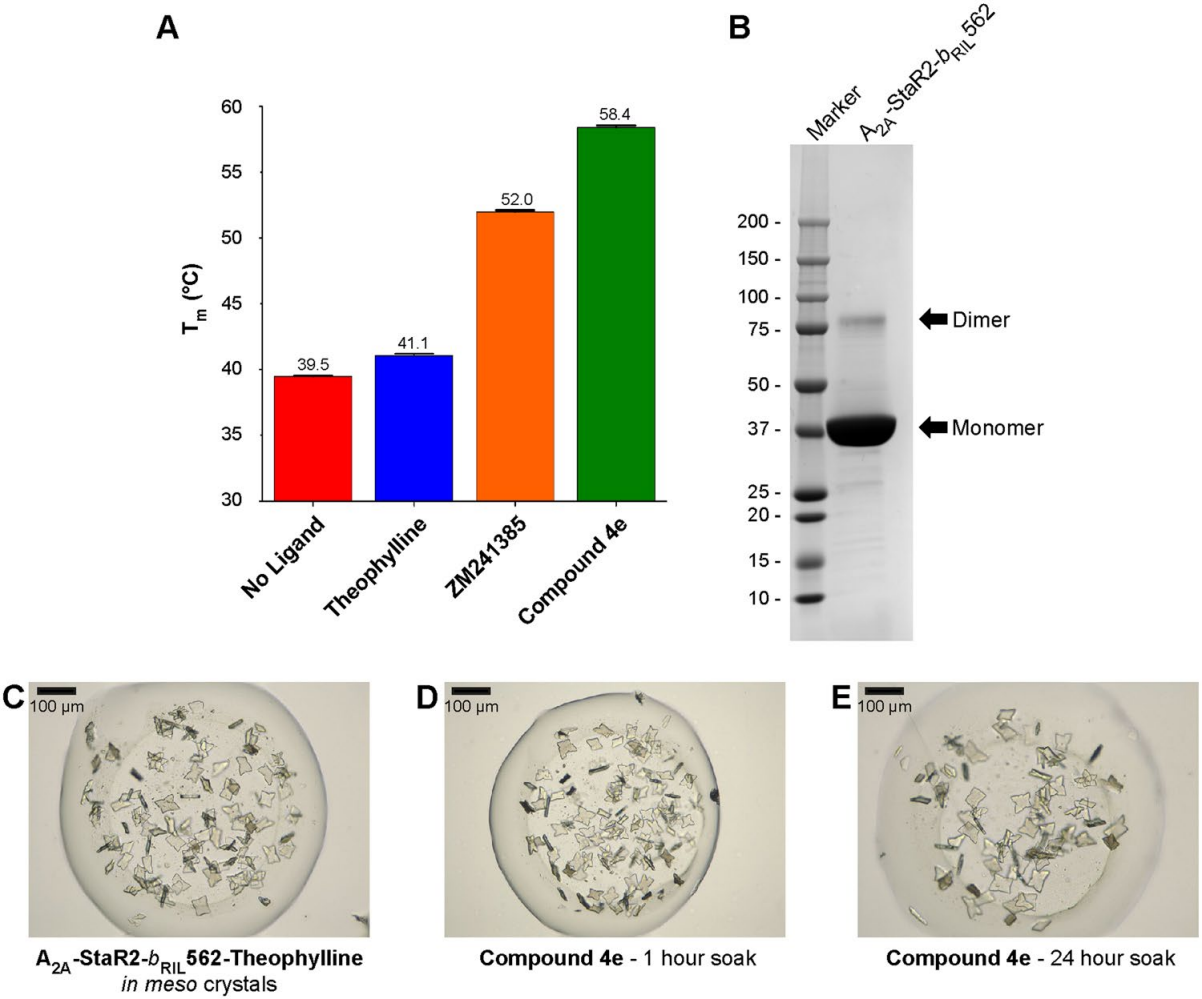
A_2A_-StaR2-*b*_RIL_562 Crystal Soaking. (**A**) Bar chart showing the melting temperature of A_2A_-StaR2-*b*_RIL_562 in its *apo* form or in the presence of theophylline, **ZM241385** or **Compound 4e**, reflecting the relative stability of each protein preparation. (**B**) SDS-PAGE of concentrated A_2A_-StaR2-*b*_RIL_562 protein prior to crystallisation. (**C**) Crystals of the A_2A_-StaR2-*b*_RIL_562-Theophylline complex. (**D**) A_2A_-StaR2-*b*_RIL_562-Theophylline crystals following a 1 hour soak in 1 mM **Compound 4e**. (**E**) A_2A_-StaR2-*b*_RIL_562-Theophylline crystals following a 24 hour soak in 1 mM **Compound 4e**.

**Figure 2:**
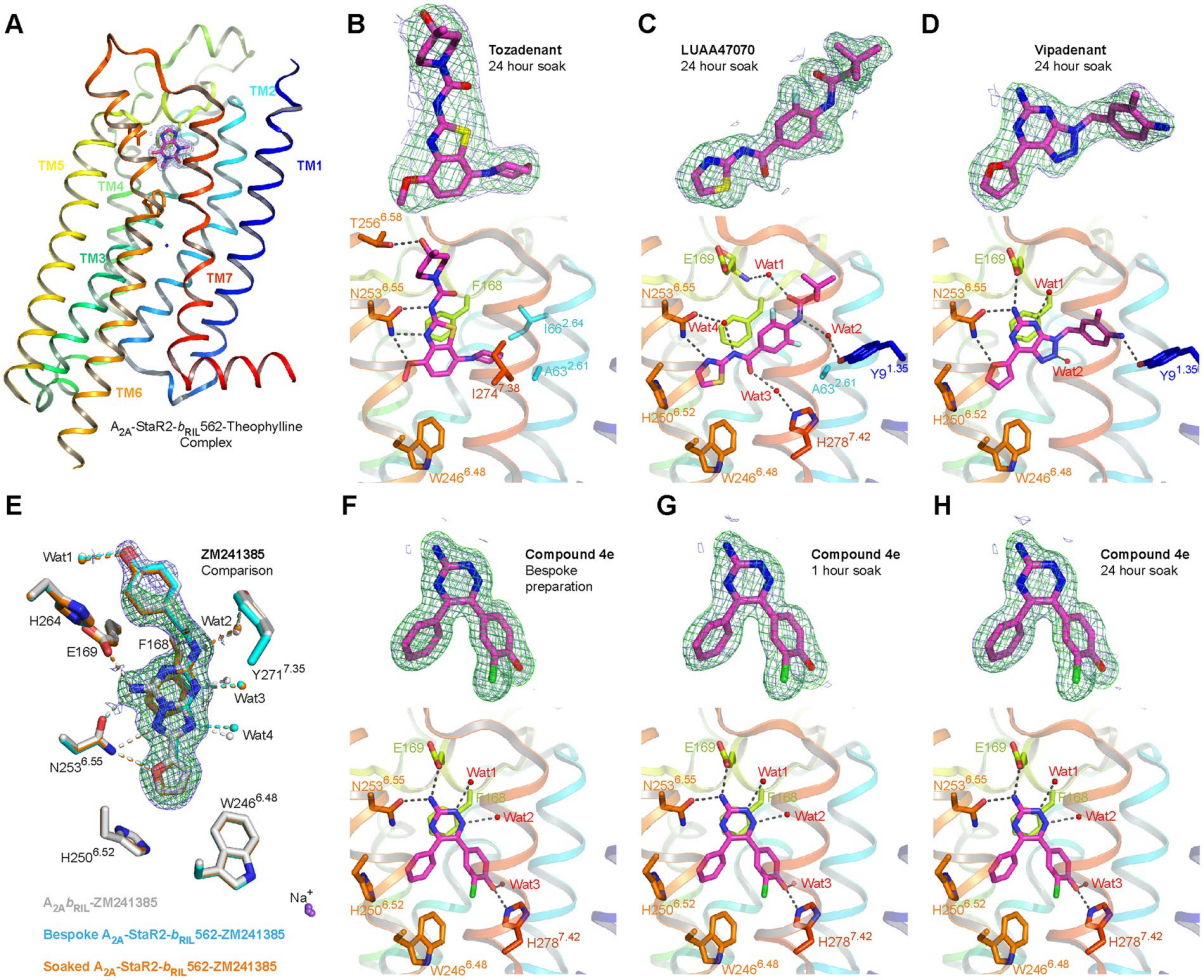
Structure of A_2A_-StaR2-*b*_RIL_562-ligand complexes. (**A**) Structure of the A_2A_-StaR2-*b*_RIL_562-Theophylline complex (PDB: 5MZJ) shown in cartoon, with helices coloured differently from blue (helix 1) to red (helix 8). Theophylline is shown as sticks within the 1.0 σ contoured 2m*Fo*-d*Fc* electron density maps (blue mesh) carved around the ligand. Interesting orthosteric binding site residues are shown as sticks. 1.0 σ contoured 2m*Fo*-d*Fc* and 3.5 σ contoured m*Fo*-d*Fc* ligand omit electron density maps (blue and green meshes respectively) reflecting the quality of ligand (purple sticks) fitting are shown in the top panel, whereas the lower panel provides interaction details between A_2A_-StaR2-*b*_RIL_562 binding site residues (sticks) with **Tozadenant** (**B**), **LUAA47070** (**C**) or **Vipadenant** (**D**). In these figures, water molecules are represented as red spheres whereas hydrogen bonding is highlighted by dotted lines. An overlay of structures of **ZM241385** in complex with A_2A_-StaR2-*b*_RIL_562 from either a bespoke preparation (PDB: 5UI4) (cyan) or from a soaking experiment (orange), and with A_2A_ *b*_RIL_562 (PDB: 4EIY) (white) depicts the high degree of conservation in positioning of orthosteric binding site residues (**E**). Residues and water molecules involved in ligand binding within a 5 Å radius are represented as sticks and as spheres respectively. Hydrogen bonds are shown as dotted lines and the 1.0 σ contoured 2m*Fo*-d*Fc* and 3.5 σ contoured m*Fo*-d*Fc* ligand omit electron density maps corresponding to **ZM241385** from the soaking experiment are represented as blue and green meshes respectively, carved around the ligand. Similarly electron density maps and interactions are shown for A_2A_-StaR2-*b*_RIL_562-**Compound 4e** generated from bespoke crystallisation (**F**) or from either 1 hour (**G**) or 24 hour (**H**) soaks of A_2A_-StaR2-*b*_RIL_562-Theophylline crystals with **Compound 4e**.

Theophylline binds to the thermostabilised receptor used for crystallisation (A_2A_-StaR2-*b*_RIL_562), with relatively low affinity (p*K*_*D*_ = 5.71), and with fast kinetics^18^, whereas potent A_2A_R-selective antagonists such as 1,2,4-triazine derivatives^19^, typically bind with higher affinity (p*K*_*D*_ > 8) and exhibit slow off-rates. Despite its low affinity for A_2A_-StaR2-*b*_RIL_562, theophylline provides some thermostabilisation to the receptor in comparison to *apo* protein (Fig. 1A). This *in meso* soaking method uses theophylline as a low affinity carrier ligand, present throughout purification, to provide crystallisation-grade A_2A_-StaR2-*b*_RIL_562 (Fig. 1B). The A_2A_-StaR2-*b*_RIL_562-Theophylline complex readily crystallises *in meso* yielding thick ~60 µm long plates (Fig. 1C), typically diffracting to 2.0 Å and containing a theophylline molecule in the A_2A_R orthosteric binding site^20^ (Fig. 2A). Crystals with theophylline have also been used previously to generate a structure with another xanthine, PSB36^20^.

The utility of the *in meso* soaking system for diverse ligands from chemical series other than xanthines was then investigated. A_2A_-StaR2-*b*_RIL_562-Theophylline crystals were soaked in mother liquor supplemented with A_2A_ antagonists **Tozadenant** ^21^ (p*K*_*D*_ = 8.4), **LUAA47070** ^22^ (p*K*_*D*_ = 6.5) or **Vipadenant** ^23^ (p*K*_*D*_ = 9.0), and their diffraction characterised. Crystals from these experiments diffracted in spacegroup *C222*_1_ to 2.0–3.1 Å resolution (Table 1). **Tozadenant**, **LUAA47070** and **Vipadenant** are all well defined in the electron density maps (Fig. 2B–D). For these ligands the basal region of the orthosteric site is delimited by Trp246^6.48^, which engages in Van der Waals contacts to the **Tozadenant** benzothiazole ring, the **LUAA47070** thiazole ring or the **Vipadenant** furan ring (Fig. 2B–D). These ligands explore different regions at the apical end of the orthosteric site. The 4-hydroxy,4-methylpiperidine moiety of **Tozadenant** sits upright on the benzothiazole ring, and hydrogen bonds to Thr256^6.58^. The N,2,2-trimethylpropanamide group of **LUAA47070** extends obliquely towards transmembrane helix 1 (TM1), and engages in water-mediated contacts with ECL2 Glu169 and Tyr9^1.35^. Further this structure shows how the experimentally defined water mediated interactions of the amide group of **LUAA47070** to both Asn253^6.55^ and His278^7.42^ contribute to this ligand binding pose. Finally, the **Vipadenant** 2-methylaniline moiety points laterally towards TM1, and is hydrogen-bonded to Tyr9^1.35^. We find that, despite adopting a range of orientations in the orthosteric binding site, ligands from different chemical series can be effectively soaked into A_2A_-StaR2-*b*_RIL_562-Theophylline crystals, and used in crystallographic structural studies to identify their binding modes. Contrary to poorly diffracting, bespoke A_2A_-StaR2-*b*_RIL_562-**Tozadenant** crystals, likely resulting from the disruption of the salt bridge between extracellular loop 2 (ECL2) Glu169 and ECL3 His264, interfering with crystal packing, co-crystals from soaking experiments yielded good quality structural data, highlighting the versatility of the *in meso* soaking system.

**Table 1:** Data collection and refinement statistics.

	Compound 4e (1 hour soak) 5OM1	Compound 4e (24 hour soak) 5OM4	Compound 4e (Bespoke) 5OLZ	Tozadenant (24 hour soak) 5OLO	Vipadenant (24 hour soak) 5OLH	LUAA47070 (24 hour soak) 5OLV	ZM241385 (24 hour soak) 5OLG
**Data collection**							
Space group	C222_1_	C222_1_	C222_1_	C222_1_	C222_1_	C222_1_	C222_1_
**Cell dimensions**							
*a*, *b*, *c* (Å)	39.54, 179.85, 140.32	39.47, 179.11, 140.03	39.37, 179.25, 140.07	39.38, 181.10, 141.77	39.40, 179.33, 141.14	39.43, 180.77, 140.90	39.45, 179.39, 139.60
*α*, *β*, *γ* (°)	90, 90, 90	90, 90, 90	90, 90, 90	90, 90, 90	90, 90, 90	90, 90, 90	90, 90, 90
Resolution (Å)	33.83–2.10 (2.16–2.10)^a^	32.92–2.00 (2.05–2.00)^a^	33.71–1.90 (1.94–1.90)^a^	38.48–3.10 (3.21–3.10)^a^	29.82–2.60 (2.72–2.60)^a^	76.08–2.00 (2.05–2.00)^a^	46.53–1.85 (1.89–1.85)^a^
*R*_pim_	0.061 (0.564)^a^	0.059 (0.635)^a^	0.040 (0.583)^a^	0.078 (0.559)^a^	0.097 (0.547)^a^	0.059 (0.624)^a^	0.068 (0.935)^a^
*I/*σ(*I*)	10.0 (1.5)^a^	10.3 (1.3)^a^	11.3 (1.4)^a^	8.3 (1.5)^a^	8.3 (1.8)^a^	8.0 (1.3)^a^	7.7 (1.0)^a^
*CC*_1/2_	0.997 (0.524)^a^	0.998 (0.439)^a^	0.999 (0.421)^a^	0.997 (0.480)^a^	0.988 (0.519)^a^	0.997 (0.420)^a^	0.994 (0.372)^a^
Completeness (%)	100.0 (100.0)^a^	98.4 (98.8)^a^	99.3 (99.6)^a^	99.9 (100.0)^a^	99.5 (98.7)^a^	98.5 (94.1)^a^	99.7 (100)^a^
Redundancy	6.6 (6.7)^a^	6.3 (6.5)^a^	4.7 (4.8)^a^	5.7 (5.9)^a^	6.9 (5.1)^a^	3.6 (3.7)^a^	6.2 (6.4)^a^
**Refinement**							
Resolution (Å)	33.83–2.10	32.92–2.00	33.71–1.90	38.48–3.10	29.82–2.60	41.68–2.00	41.31–1.86
No. reflections	56388	63810	74939	9645	15827	34169	41376
*R*_work_/*R*_free_	0.1882/0.2097	0.1831/0.2049	0.1727/0.1963	0.1987/0.2448	0.2000/0.2487	0.1801/0.2081	0.1921/0.2332
**No. atoms**							
Protein	3082	3083	3104	2983	3047	3083	3097
Ligand	21	21	21	28	24	24	25
Solvent	687	727	684	397	488	607	597
***B*** **factors**							
Protein	40.55	39.22	45.33	80.98	43.21	40.19	36.81
Ligand	19.40	17.37	20.13	49.00	21.26	18.28	27.17
Solvent	55.49	56.88	59.69	87.00	51.60	51.83	49.20
**R.m.s. deviations**							
Bond lengths (Å)	0.002	0.003	0.004	0.002	0.003	0.009	0.014
Bond angles (°)	0.92	0.93	0.99	0.74	0.75	1.19	1.54

^a^Values in parentheses are for highest-resolution shell. All data presented above were collected from single crystals except for the A_2A_-StaR2-*b*_RIL_562-**Vipadenant** complex, where data was merged from three different crystals.

The validity of structural results obtained by the *in meso* soaking method was checked using **ZM241385** ^24^ (p*K*_*D*_ = 8.6), a well-characterised A_2A_R antagonist that increases A_2A_-StaR2-*b*_RIL_562 stability by ~12 °C (Fig. 1A). The crystal structure of the receptor in complex with ZM241385 resulting from *in meso* soaking, was compared with similar complexes obtained from bespoke crystallisation setups using either A_2A_-StaR2-*b*_RIL_562^25^ or A_2A_-*b*_RIL_562^26^ (Fig. 2E). Overlaying these structures shows a remarkably similar structural conformation of residues in the orthosteric located within 5 Å of the ligand with an all atom r.m.s.d. of only 0.074 Å (soaked v/s bespoke A_2A_-StaR2-*b*_RIL_562 (PDB: 5IU4)) or 0.118 Å (soaked v/s bespoke A_2A_-*b*_RIL_562 (PDB: 4EIY)) (Fig. 2E). Such a high degree of structural conservation across different crystallisation methods (and A_2A_R constructs) benchmarks and underlines the robustness of the *in meso* soaking system described here.

To determine the feasibility of using the *in meso* soaking method system to support optimisation of novel A_2A_R antagonists for drug discovery, **Compound 4e**, a 1,2,4-triazine derivative^19^, was investigated. **Compound 4e** is a low nanomolar affinity ligand (p*K*_*D*_ = 9.6) for A_2A_R and increases A_2A_-StaR2-*b*_RIL_562 stability by ~19 °C when compared to *apo* protein (Fig. 1A) and co-crystals were generated using either a bespoke protein preparation or by soaking A_2A_-StaR2-*b*_RIL_562-Theophylline crystals in mother liquor supplemented with **Compound 4e** for 1 or 24 hours (Fig. 1D,E). Crystal morphology remained unchanged regardless of soaking times (Fig. 1D,E) and crystals from these three experiments diffracted to 1.9–2.1 Å in spacegroup *C222*_1_. Structures generated from bespoke crystallisation or from the soaking **experiments** were essentially equivalent (r.m.s.d ~0.1 Å over 297 residues). **Compound 4e** was well defined in electron density maps from the resultant three co-structures and binds in the same orientation in the orthosteric site (Fig. 2F–H), displaying similar *B factors* (17.8–19.8 Å^2^) (Table 1). **Compound 4e** sits lower in the orthosteric site than theophylline, with the triazine ring π stacking against Phe168 from ECL2, while also engaging in polar contacts with an extensive water network. The amine moiety on the triazine ring is further hydrogen-bonded to ECL2 Glu169 and Asn253^6.55^, whereas the hydroxyl group on the chlorophenol ring makes a hydrogen bond with His278^7.43^. In the basal region of the orthosteric site, the ligand benzyl ring makes Van der Waals interactions with Trp246^6.48^.

A pairwise comparison of residues located within 5 Å of all the different liganded structures presented here demonstrates all atom r.m.s.d. values ranging from 0.48 Å (between the A_2A_-StaR2-*b*_RIL_562-**Compound 4e** and -**LUAA47070** structures) to 1.05 Å (between the A_2A_-StaR2-*b*_RIL_562-**ZM241385** and -**Tozadenant** structures). Altogether, most of the mobility stems from Tyr271^7.35^, involved in water-mediated interactions with **ZM241385**, and from Glu169 in ECL2 and His264 which adopt different rotamer orientations in the A_2A_-StaR2-*b*_RIL_562-**Tozadenant** structure compared to the other ligand complexes.

In drug development, high-throughput X-ray crystallography expedites the elaboration of novel hits into lead compounds and drug candidates by providing multiple high resolution views of ligand-receptor complexes, which are key for understanding critical intermolecular interactions alongside interpretation of ligand-induced receptor conformational changes^27^. The accelerated availability of multiple receptor-ligand complexes provides a data-rich starting point for SBDD and medicinal chemistry^28^ which, when correlated with *in vitro* biological activity, allows rapid incorporation of molecular modifications towards increasing ligand affinity for the binding site or improvement of their absorption, distribution, metabolism, excretion and toxicity (ADMET) properties.

We have demonstrated that an *in meso* ligand soaking methology can rapidly and efficiently yield multiple high-resolution co-crystal structures from a diverse set of ligands in complex with a given GPCR. Such soaking techniques have also been employed *in-house* for other discovery projects. The method described here has general applicability to further discovery campaigns with stabilised membrane proteins using LCP crystallisation setups, provided high quality crystals exist for the target in complex with low affinity stabilising carrier ligands with fast off-rates.

## Methods

### StaR generation

The thermostabilisation of the human A_2A_ receptor (resulting in A_2A_-StaR2) using a mutagenesis approach^8^, has been previously described^29^.

### Expression, membrane preparation and protein purification

The A_2A_-StaR2-*b*_RIL_562 construct has been described previously^25^ and harbours eight thermostabilising mutations (A54L^2.52^, T88A^3.36^, R107A^3.55^, K122A^4.43^, L202A^5.63^, L235A^6.37^, V239A^6.41^ and S277A^7.42^) as well as a mutation to remove a glycosylation site (N154A). The construct further comprises an Apocytochrome *b*_RIL_562 fusion between transmembrane helices 5 and 6 and a C-terminal decahistidine tag. The receptor was expressed using the Bac to Bac Expression System (Invitrogen) in *Trichoplusa ni* Tni PRO cells using ESF 921 medium (Expression Systems) supplemented with 5% (v/v) fetal bovine serum (Sigma-Aldrich) and 1% (v/v) Penicillin/Streptomycin (PAA Laboratories). Cells were infected at a density of 2.6 × 10^6^ cells/ml with virus at an approximate multiplicity of infection of 1. Cultures were grown at 27 °C with constant shaking and harvested by centrifugation 48 hours post infection. All subsequent protein protein purification steps were carried out at 4 °C unless otherwise stated.

For each protein preparation, cells from 2 L cultures were resuspended in 40 mM TRIS buffer at pH 7.6 supplemented by 1 mM EDTA and Complete EDTA-free protease inhibitor cocktail tablets (Roche). Cells were disrupted at ~15 000 psi using a microfluidizer (Processor M-110L Pneumatic, Microfluidics). Membranes pelleted by ultra-centrifugation at 200 000 g for 50 minutes, were subjected to a high salt wash in a buffer containing 40 mM Tris pH 7.6, 1 M NaCl and Complete EDTA-free protease inhibitor cocktail tablets, before they were centrifuged at 200,000 g for 50 minutes. Washed membranes were resuspended in 50 mL 40 mM Tris pH 7.6 supplemented with Complete EDTA-free protease inhibitor cocktail tablets and stored at −80 °C until further use.

Protein preparations intended for soaking experiments were carried out in the presence of theophylline whereas the bespoke preparation of A_2A_-StaR2-*b*_RIL_562 in complex with **Compound 4e** was done in the presence of 5 µM ligand.

Membranes were thawed, resuspended in a total volume of 150 ml with 40 mM Tris–HCl pH 7.6, Complete EDTA-free protease inhibitor cocktail tablets (Roche), 3 mM theophylline (Sigma Aldrich) (or 5 µM **Compound 4e**), and incubated for 2 hours at room temperature. Membranes were then solubilized by addition of 1.5% n-Decyl-β-D-maltopyranoside (DM, Anatrace), and incubation for 2 hours at 4 °C, followed by centrifugation at 145 000 g for 60 min to harvest solubilised material.

The solubilised material was applied to a 5 ml Ni-NTA (nickel-nitrilotriacetic acid) Superflow cartridge (Qiagen) pre-equilibrated in 40 mM Tris pH 7.4, 200 mM NaCl, 0.15% DM, 1 mM theophylline (or 5 µM **Compound 4e**). The column was washed with 25 column volumes of buffer 40 mM Tris pH 7.4, 200 mM NaCl, 0.15% DM, 70 mM imidazole, 1 mM theophylline (or 5 µM **Compound 4e**) and then the protein was eluted with 40 mM Tris pH 7.4, 200 mM NaCl, 0.15% DM, 280 mM imidazole, 1 mM theophylline (or 5 µM **Compound 4e**).

Collected fractions were analyzed by SDS PAGE and fractions containing A_2a_-StaR2-*b*_RIL_562 were pooled and concentrated using an Amicon Ultra Ultracell 50 K ultrafiltration membrane to a final volume of ~800 µl. The protein sample was ultra-centrifuged at 436 000 g for 10 minutes before being applied to a Superdex200 size exclusion column (GE Healthcare) pre-equilibrated with 40 mM Tris pH 7.4, 200 mM NaCl, 0.15% DM, 1 mM theophylline (or 5 µM **Compound 4e**). Eluted fractions containing the protein were analyzed by SDS PAGE, pooled and concentrated to ~35 mg/ml using an Amicon Ultra Ultracell 50 K ultrafiltration membrane and subjected to an ultra-centrifugation at 436 000 g prior to crystallisation. Protein concentrations were measured using the DC assay (Bio-Rad), and confirmed using quantitative amino acid analysis.

### Thermal unfolding experiments

A_2A_-StaR2-*b*_RIL_562 purified in DM in the presence of 500 µM theophylline was used for thermal unfolding experiments. The protein was diluted in 40 mM Tris pH 7.4, 200 mM NaCl, 0.15% DM to a final concentration of 0.2 mg/ml. Following heavy dilution (~70-fold) of the protein in a buffer without ligand, the sample was considered to be in an *apo*-like state. Samples were supplemented with the respective ligands to a final concentration of 50 µM, with a final DMSO concentration of 5% (v/v). The control sample was supplemented with DMSO to a final concentration of 5% (v/v). Samples were incubated 30 minutes on ice before being loaded into UV capillaries (NanoTemper Technologies) and experiments were carried out using the Prometheus NT.48. The temperature gradient was set to an +1 °C/min from 20 °C to 90 °C. Protein unfolding was measured by detecting the temperature-dependent change in tryptophan fluorescence at emission wavelengths of 330 and 350 nm. The experiment was repeated four times and data analysed with the one-way analysis of variance (ANOVA) with Dunnett’s post-test. Tm values obtained for the three ligands are statistically different from the control sample with p < 0.001.

### Crystallisation

The A_2A_-StaR2-*b*_RIL_562 in complex with either theophylline or **Compound 4e** was crystallized in lipidic cubic phase at 20 °C. Concentrated protein was mixed with monoolein (Nu-Chek) supplemented with 10% (w/w) cholesterol (Sigma Aldrich) and 10 µM theophylline (or 5 µM **Compound 4e**) using the twin-syringe method^30^. The final protein:lipid ratio was 40:60 (w/w). 40 nl boli were dispensed onto 96-well Laminex Glass Bases (Molecular Dimensions ltd) using a Mosquito LCP crystallization robot (TTP Labtech) and overlaid with 800 nL precipitant solution. Glass bases were sealed using Laminex Film covers (Molecular Dimensions Ltd). 60–80 µm long plate-shaped crystals grew within 2 weeks in 0.l M tri-sodium citrate pH 5.3–5.4, 0.05 M sodium thiocyanate, 29–32% PEG400, 2% (v/v) 2,5-hexanediol and 0.5 mM theophylline (or 5 µM **Compound 4e**).

### *In meso* soaking and crystal harvesting

For soaking experiments, incisions were made into the Laminex cover over base wells containing crystals identified for harvesting and these wells were flooded with 10 µL motherliquor supplemented by 1 mM ligand. The crystals are soaked in motherliquor with a final ligand concentration of 925 µM, and a final theophylline concentration of 74 µM. Flooded wells were then re-sealed using Crystal Clear Sealing Tape (Hampton Research), and plates were incubated for 1 hour or 24 hours at 20 °C. Single crystals were mounted in LithoLoops (Molecular Dimensions Ltd) and flash-frozen in liquid nitrogen without the addition of further cryoprotectant.

### Diffraction data collection and processing

X-ray diffraction data were measured on a Pilatus 6 M detector at beamline I24 (Diamond Light Source) using a 6 × 9 μm beam size of for crystals of A_2A_-StaR2-*b*_RIL_562 in complex with **Compound 4e**, **Tozadenant** or **LUAA47070**. Complete datasets were acquired from a single crystal for each of these complexes at wavelengths 0.96857 Å (**Compound 4e** and **LUAA47070**) or 0.96862 Å (**Tozadenant**), using an unattenuated beam and 0.2° oscillation per frame, with an exposure of 0.1 second per degree of oscillation. Diffraction data for the A_2A_-StaR2-*b*_RIL_562-**Vipadenant** complex were acquired from 3 different crystals on an Eiger 16 M detector at beamline X06SA (Swiss Light Source) at a wavelength of 1 Å, using 10% beam transmission and 0.1° oscillation per frame, with an exposure of 1 second per degree of oscillation. The A_2A_-StaR2-*b*_RIL_562-**ZM241385** data was collected from a single crystal on an Eiger 16 M detector at beamline × 06SA at a wavelength of 1 Å, using 20% beam transmission and 0.25° oscillation per frame, with an exposure of 0.24 second per degree of oscillation. Data from individual crystals were integrated using *XDS* ^31^, merged and scaled using *AIMLESS* ^32^ from the CCP4 suite^33^. Data collection statistics are reported in Table 1.

### Structure solution and refinement

The structures of the different A_2A_-StaR2-*b*_RIL_562-ligand complexes were solved by molecular replacement (MR) with *Phaser* ^34^ using the A_2A_-StaR2-*b*_RIL_562-theophylline complex structure^20^ as the search model (PDB code: 5MZJ). Iterative rounds of model refinement performed using *phenix.refine* ^35^, were interspersed with manual model building in *COOT* ^36^. Both xray and B-factor restraint weights were optimised in *phenix.refine*, and 2 TLS groups corresponding to the receptor and to the *b*_RIL_562 respectively were defined during refinement. Refinement was with positional and individual isotropic B-factor refinement. The final models were validated using MolProbity^37^. The final refinement statistics are presented in Table 1. Structure figures were generated using PyMOL^38^. The three structures of the A_2A_-StaR2-*b*_RIL_562-**Compound 4e** reported here generated from crystals grown using LCP, are comparable with the previously reported A_2A_-StaR2-**Compound 4e** structure (PDB: 3UZC)^19^ solved from crystals grown using the vapour diffusion technique, with an all atom r.m.s.d. of 0.91 Å over 276 residues.

### Data Availability Statement

The data that support the findings of this study are available from the corresponding author upon reasonable request. Co-ordinates and structure factors have been deposited in the Protein Data Bank under the accession codes 5OM1, 5OM4, 5OLZ, 5OLV, 5OLO, 5OLH and 5OLG.
